# Case Report: An association of the gubernacular canal, supernumerary tooth and odontoma with an impacted canine on cone-beam computed tomography

**DOI:** 10.12688/f1000research.26627.2

**Published:** 2021-02-26

**Authors:** Lubna K. Elsayed, Sara M. El Khateeb, Suzan A. Alzahrani, Shatha Subhi ALHarthi, Raidan Ba-Hattab

**Affiliations:** 1 Oral Surgery Department, Suez Canal University, Ismailia, Egypt; 2 Basic Dental Sciences Department, College of Dentistry, Princess Nourah bint Abdulrahman University, Riyadh, Saudi Arabia; 3 Dental Intern, College of Dentistry, Princess Nourah bint Abdulrahman University, Riyadh, Saudi Arabia; 4 Preventive Dental Sciences Department, College of Dentistry, Princess Nourah Bint Abdulrahman University, Riyadh, Saudi Arabia; 5 College of Dental Medicine, Qatar University, Doha, Qatar

**Keywords:** Gubernacular canal, Compound Odontoma, Supernumerary teeth, Cone Beam CT, Impacted Canine

## Abstract

This report describes a clinical case of asymptomatic compound odontoma in the anterior left side of the maxilla associated with an impacted canine and supernumerary tooth with a gubernacular canal of a 47- year-old female with no relevant medical history. Cone-beam computed tomography (CBCT) was performed for precise three-dimensional localization of each structure and assessment of their spatial relationship with the associated structures before surgery. The treatment protocol involved surgical enucleation of the odontoma and open extraction of both impacted and supernumerary teeth. The patient had uneventful healing and proceeded with the prosthodontic treatment plan.

The dentist should be aware of the probability of a close relationship between the development of odontoma and presence of the gubernacular tract, which could be used as a future radiographic diagnostic criterion of an odontoma. Also, we recommend that more studies be performed in this field to deeply analyze the imaging characteristics of GT and its spatial association with various pathological lesions in the future.

## Introduction

Odontoma is defined as a benign odontogenic tumor containing enamel, dentin and cementum, and are classified by the World Health Organization into two main types: compound and complex ^1, 2^. Compound odontoma consists of a tumorlike malformation (hamartoma) with varying numbers of tooth-like elements (odontoids). The complex odontoma consists of a tumour-like malformation (hamartoma) in which the enamel and dentin, and sometimes cementum, are present ^3^. Pathogenesis of odontoma is still unclear, although some etiologic factors have been suggested such as trauma during primary dentition, genetic factors, and chronic inflammation ^4^. They are usually small, asymptomatic and discovered through radiographic examination when patients present with a missing permanent tooth ^5, 6^.

Odontomas may cause disturbances in the eruption of teeth such as impaction, delayed eruption, or retention of deciduous or permanent teeth, despite these critical disturbances only a few patients have been described ^7^

An impacted tooth is one that fails to fully erupt into the dental arch within the usual range of expected time. The tooth becomes impacted because abnormal tooth orientation, adjacent teeth, dense overlying bone, excessive soft tissue, or a genetic abnormality prevents eruption ^8^. Exceptionally, it can be associated with the supernumerary teeth or an odontoma ^1^.

The gubernacular cord is a structure composed of conjunctive tissue that connects the tooth follicle to the overlying gingiva, this cord guides or directs the course of the tooth eruption. A canal is formed within the bone by the osteoblastic activity to contain the gubernacular cord which is named gubernacular canal ^9– 11^

We present a case of an impacted upper canine with compound odontoma and a supernumerary tooth accompanied by gubernacular canal, where we utilized cone-beam computed tomography (CBCT) to locate each structure precisely prior to surgical treatment.

## Case report

**Figure 1.**
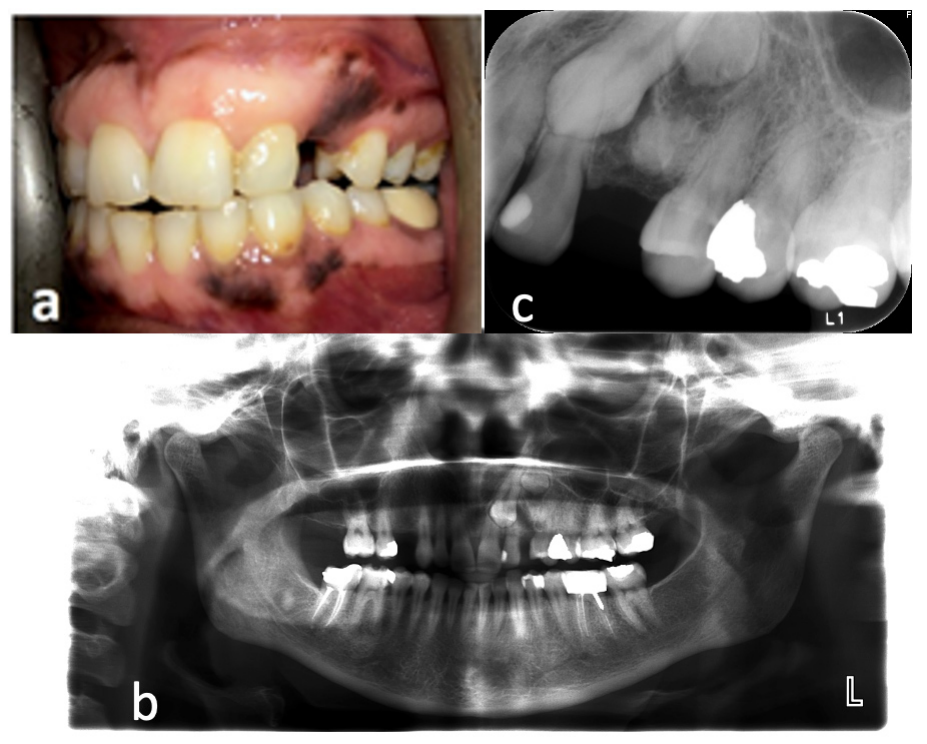
**a**) Preoperative clinical intraoral photo showing buccal bulging opposite to the area of impacted left maxillary canine. **b**) preoperative panoramic radiograph showing impacted left maxillary canines and two radiopaque masses distal to it. **c**) preoperative periapical radiograph showing impacted canine, supernumerary tooth and radiopaque mass.

A 47-year old female – of African origin - presented to the dental clinic to treat multiple carious teeth, and to replace multiple missing teeth. On Intraoral clinical examination, both the maxillary canines were missing as well as the third molars, and there was a bulge on the buccal cortex of the missing maxillary left canine, which was asymptomatic ( Figure 1a). The patient’s medical history showed no previous incidence of dental/maxillofacial trauma or infections. She was referred to the Oral and Maxillofacial Surgery clinic for management. Panoramic radiograph revealed the presence of an impacted maxillary left canine and two small radiopaque masses distal to the impacted canine ( Figure 1b) and the periapical radiograph showed that the apical mass is a supernumerary tooth and the coronal mass is a rounded denticle like mass ( Figure 1c).

**Figure 2.**
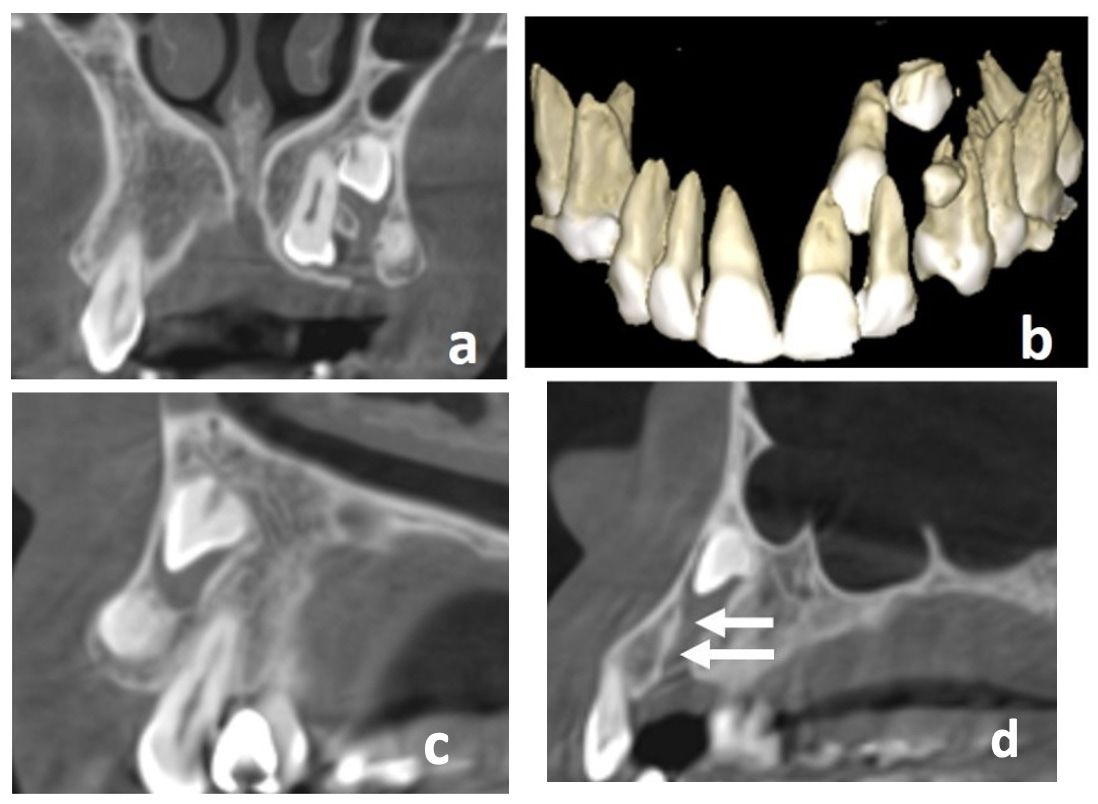
**a**) Coronal cone-beam computed tomography (CBCT) scan showing impacted left maxillary canine, supernumerary tooth and odontoma with continuous follicular space **b**) Three-dimensional CBCT reconstruction showing palatal impacted maxillary left canine together with supernumerary tooth and odontoma **c**) Sagittal CBCT scan showing odontoma occlusal to supernumerary tooth **d**) Sagittal CBCT scan showing the proposed gubernacular canal (white arrow).

 CBCT showed that tooth #23 was palatal impacted between teeth #21 and #22. There was a very small well defined rounded denticle like mass positioned distal to tooth #22 and coronal to the impacted supernumerary tooth (crown only), preventing its eruption. ( Figure 2a, b, c).

The sagittal CBCT slice showed radiographic evidence of continuity of the follicular space of the supernumerary tooth along the bone up to its most inferior part at the alveolar ridge. This was determined to most likely be the gubernacular canal as it followed its possible eruption pathway through the bone. However, the denticle like structure was located inside this pathway and prevented the eruption of the supernumerary tooth. ( Figure 2d)

The most probable differential diagnosis would be a compound odontoma because of its denticle like density, organization, and coronal position.

Intraoral surgery was planned under local anaesthesia, based on the clinical, radiographic findings and multidisciplinary consultation diagnosis of the radiopaque mass as a compound odontoma.

**Figure 3.**
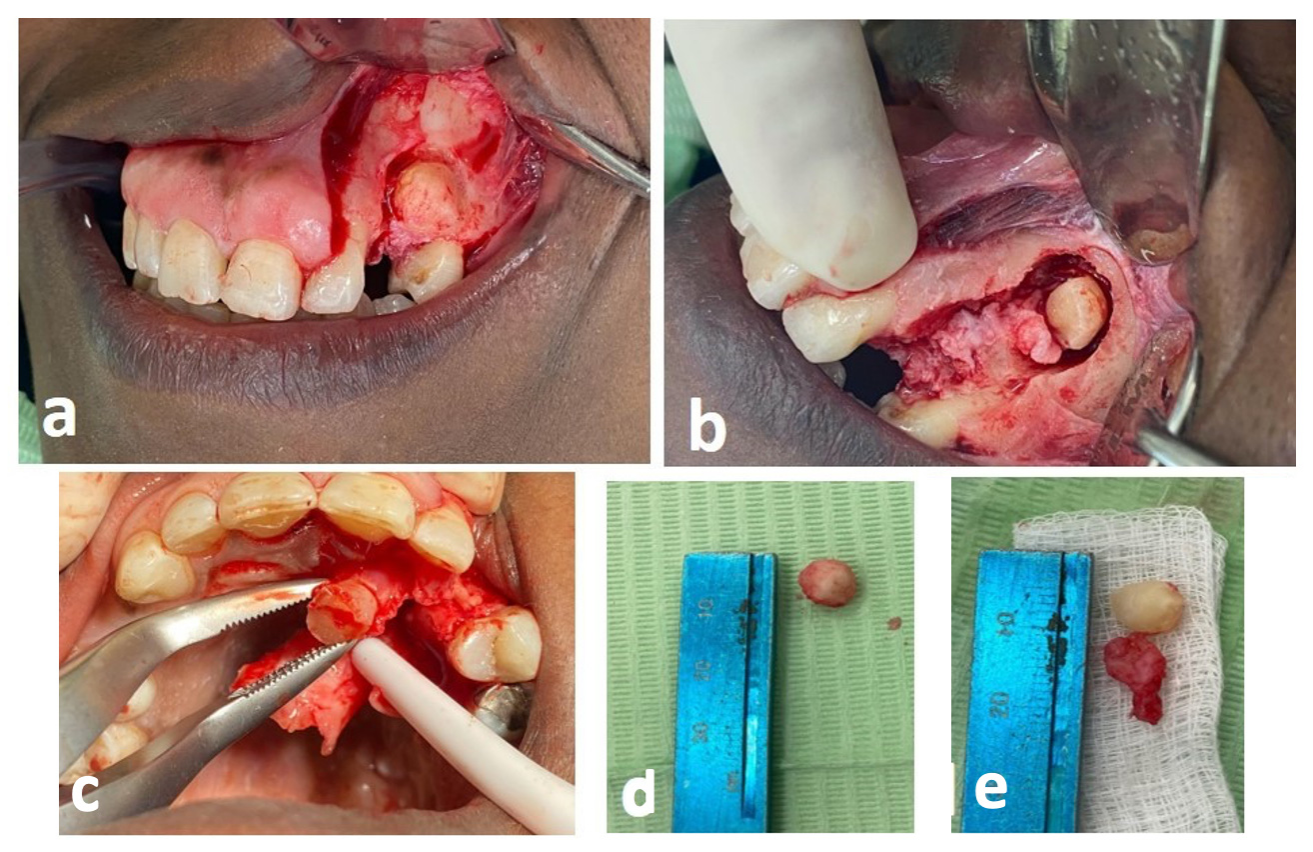
Intra-operative clinical picture showing surgical procedures to extract impacted maxillary left canine, supernumerary and odontoma **a**) Labial approach to expose the odontoma. **b**) Exposing the supernumerary tooth **c**) Palatal approach to extract the impacted maxillary left canine **d**& **e**) Measuring size of odontoma, supernumerary tooth and follicular tissue.

Local infiltration of the area was performed, a full-thickness mucoperiosteal buccal flap was raised to expose both the mass and supernumerary tooth and soft tissue in between, a palatal flap was raised to expose the impacted canine. The cortical bone was removed utilizing rotary instruments accompanied by normal saline irrigation to minimize heat generation. The calcified mass was identified and ditched all around and then elevated with a Coupland elevator. The incompletely formed supernumerary tooth along with soft tissue attached to it was extracted using straight forceps, and for identification, the soft tissue was tagged with a black silk suture. By access from the palatal region, the impacted canine was sectioned horizontally and extracted ( Figure 3 a, b, c), then the whole area was irrigated using normal saline and the flap was stitched back to its original position. A pre-surgical fabricated acrylic stent was placed to prevent falling of the palatal flap and to promote healing. Post-operative analgesia was prescribed in the form of acetaminophen 500mg every 6 hours. Clinical examination was performed 3 months post-surgical; patient was symptoms free and had uneventful recovery.

Macroscopically, the calcified mass measured 0.7 × 0.5 × 0.3 cm ( Figure 3 d), while the soft tissue mass measured 1.9 × 0.4 × 0.3 cm ( Figure 3e). Both specimens were placed in 10% buffered formalin for histopathological/histological examination and final diagnosis.

**Figure 4.**
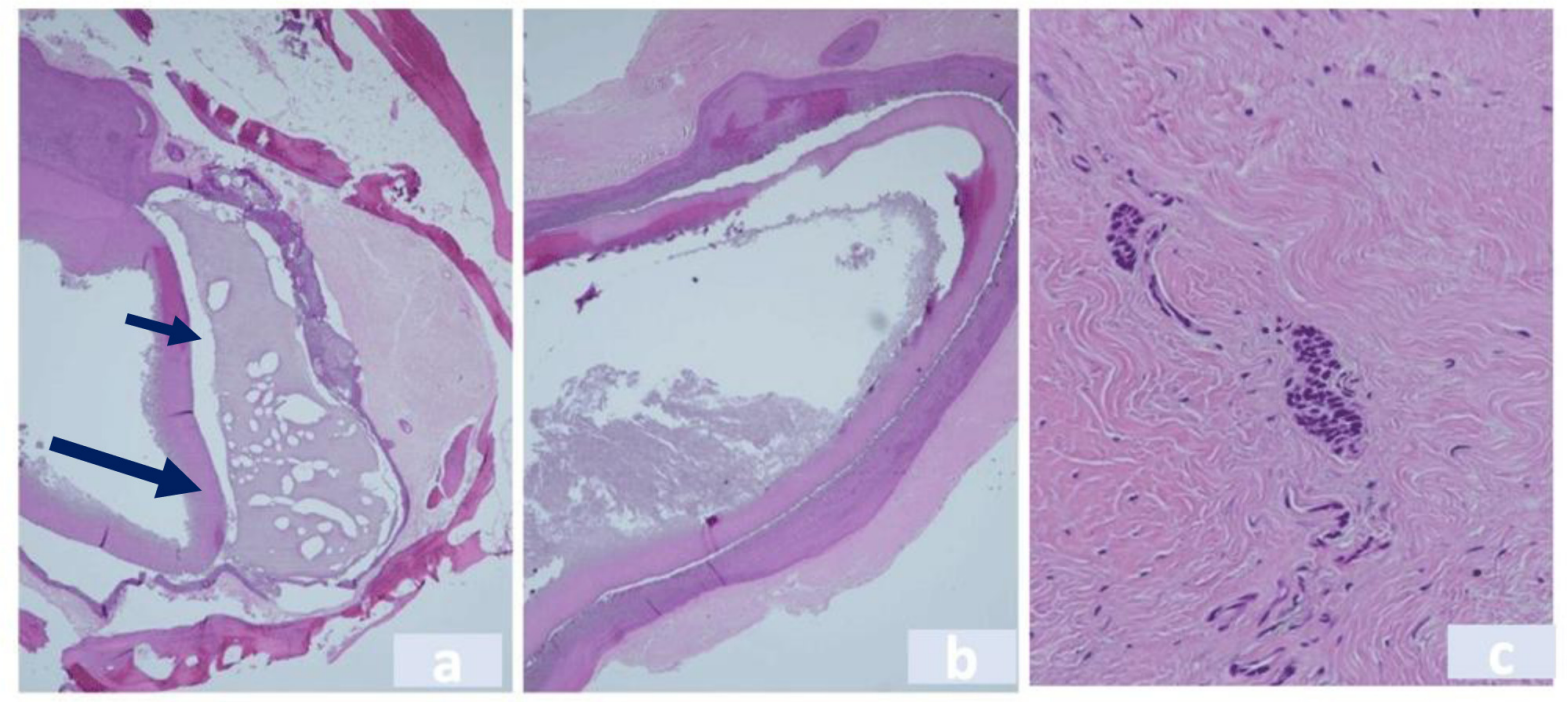
Histopathologic examination of the excised mass showed a tooth-like structure with dentin (long arrow), and enamel matrix (short arrow) confirming the diagnosis of compound odontoma ( Figure 4 a,b). Histological examination for the soft tissue mass showed odontogenic epithelial lamina arranged in islands and cords and surrounded by collagenous connective tissue which confirms the diagnosis of gubernacular tissue ( Figure 4c) (100X).

Histopathologic examination of the excised mass showed a tooth-like structure with dentin, and some enamel matrix confirming the diagnosis of compound odontoma ( Figure 4 a,b). Histological examination for the soft tissue mass showed epithelial lamina surrounded by collagenous connective tissue which confirms the diagnosis of gubernacular tissue ( Figure 4c).

## Discussion

Odontoma is a benign tumour that is usually asymptomatic, diagnosed clinically through routine radiographic examinations, or when investigating other events such as the delayed eruption of permanent teeth, or ectopic position of teeth ^12– 14^, as is the case in our findings.

Odontomas are mostly located at the anterior maxilla and associated primarily with permanent teeth, although they can also be associated with deciduous teeth and also can be associated with some complications such as impacted teeth, as in the present case, maxillary sinus complications ^15– 18^ or/and cystic association ^19^. Due to its rare recurrence, conservative surgical excision is the treatment of choice for odontomas ^8, 20^

Supernumerary teeth occur most frequently at the anterior midline, causing malposition of neighboring teeth or preventing their eruption ^1, 6, 21^. Occasionally, supernumerary teeth may be associated with some developmental syndromes like, cleidocranial dysostosis, Down’s syndrome, Apert syndrome and Gardener’s syndrome ^22^

In the present case, the supernumerary tooth was non-syndromic, incompletely formed, and it was extracted along with the impacted canine and odontoma.

Panoramic and periapical radiographs are conventional two-dimensional radiographic techniques which are widely available and are frequently used for multiple diagnostic purposes like detection of impaction, odontoma, bone loss and various intraosseous lesions. Nevertheless, these radiographs have various limitations in revealing the buccolingual relationship, and presence of superimposition, also having geometric inaccuracy ^23^. In the current case, we primarily detect the presence of impaction of a supernumerary tooth and odontoma through panoramic and periapical radiograph.

CBCT is an advanced 3D imaging modality that offers precise localization and detection of the spatial relationships of any dental structure with the surroundings, also it lessens the radiation dose in comparison with conventional CT and delivers high spatial resolution ^24^. In our case, we used CBCT for 3D localization of the odontoma, impacted canine and supernumerary tooth before surgical removal ^24, 25^.

The role of gubernacular cord (GC) and the canal in tooth eruption is not clear, although it has been suggested that it may have a central role in inducing normal tooth eruption as it constitutes a pathway from the dental follicle to the gingiva for the eruption of permanent teeth ^11^. Oda M. *et al.* ^26^ suggested that the presence of and contact with the gubernacular tract (GT) should be added as a characteristic CT finding of some types odontogenic masses including dentigerous cysts, calcifying odontogenic cysts (Gorlin Cyst), odontomas, Adenomatoid Odontogenic Tumor and perhaps others. Moreover, they concluded that dentists should pay more attention to the association between the GT and odontogenic masses ^26, 27^. Our case demonstrated an association between the GT and the odontoma where the odontoma was inside the GT of the supernumerary tooth with the same spatial relationship in agreement with Oda M. *et al.* ^26^ which reports that the majority of odontoma cases (about 70%) detected with CBCT were inside the GT of the unerupted teeth ( Figure 2d). Also, Oda M. *et al.* ^26^ reported that the presence of GT helped in differentiating complex odontomas from bone dysplasia and cemento-ossifying fibromas.

Moreover, Ide F *et al.* 2011 ^28^ stated that the Gubernacular cord may act as a source of epithelial remnants of the dental lamina which could be the basis of the development of some odontogenic tumors and/or cysts, Thus, the identification of imaging features of GT in CBCT may assist in the diagnosis of odontogenic cysts and tumors.

Furthermore, oda *et al.* ^29^ found that the continuity of the pathological lesion with the GT could be a characteristic differentiating imaging feature between some types of odontogenic and non-odontogenic tumors as this continuity was detected in CT of almost all of the odontogenic lesions (93.7%) in this study.

This was in harmony with our study, where during surgery, it was apparent that the GT was contiguous with the supernumerary follicle, suggesting that the GT was guiding the eruption of the supernumerary tooth, but the development of the odontoma prevented its eruption. Gaêta-Araujo H *et al.* reported that the most communal attachment site of GC was to the occlusal side of the dental sac of the unerupted tooth (93.2%) and was classified as a usual attachment. These findings were in agreement with our case, where we also found the GT to be attached to the occlusal aspect of the dental sac of the supernumerary tooth ^27^.

## Conclusion

The dentist should be aware of the probability of a close relationship between the development of odontoma and presence of the gubernacular tract, which could be used as a future radiographic diagnostic criterion of an odontoma. Also, we recommend that more studies to be performed in this field to deeply analyze imaging characteristics of GT and its spatial association with various pathological lesions in the future.

## Consent

Written informed consent for publication of their clinical details and clinical images was obtained from the patient.

## Data availability

### Underlying data

All data underlying the results are available as part of the article and no additional source data are required.
