# Supplementary material for: Case Report: An association of the gubernacular canal, supernumerary tooth and odontoma with an impacted canine on cone-beam computed tomography
Source: F1000Res. 2021 Feb 26;9:1204. Originally published 2020 Oct 6. [Version 2] doi: 10.12688/f1000research.26627.2 (PMC7863995; doi:10.12688/f1000research.26627.2)
Supplement: Peer review history

# Peer review: Reviewer response for version 2

**DOI:** 10.5256/f1000research.54718.r80302

**Published:** 2021-03-15

**Authors:** Prashanth Panta

**Affiliations:** 1 Department of Oral Medicine and Radiology, MNR Dental College and Hospital, Sangareddy, Telangana, India

The revisions have addressed all posted queries, and the improved version is a good read.

Are enough details provided of any physical examination and diagnostic tests, treatment given and outcomes?

Yes

Is the case presented with sufficient detail to be useful for other practitioners?

Yes

Is sufficient discussion included of the importance of the findings and their relevance to future understanding of disease processes, diagnosis or treatment?

Yes

Is the background of the case’s history and progression described in sufficient detail?

Yes

Reviewer Expertise:

Oral medicine and radiology.

I confirm that I have read this submission and believe that I have an appropriate level of expertise to confirm that it is of an acceptable scientific standard.

# Peer review: Reviewer response for version 2

**DOI:** 10.5256/f1000research.54718.r80301

**Published:** 2021-02-26

**Authors:** Ronell Bologna-Molina

**Affiliations:** 1 Molecular Pathology Area, School of Dentistry, Universidad de la República, Montevideo, Uruguay; 2 Universidad Juárez del Estado de Durango, Durango, Mexico

I thank the authors for their effort and for accepting suggestions to improve the article.

Are enough details provided of any physical examination and diagnostic tests, treatment given and outcomes?

Yes

Is the case presented with sufficient detail to be useful for other practitioners?

Partly

Is sufficient discussion included of the importance of the findings and their relevance to future understanding of disease processes, diagnosis or treatment?

Partly

Is the background of the case’s history and progression described in sufficient detail?

Yes

Reviewer Expertise:

Oral Pathology, Histopathology, Oral Medicine

I confirm that I have read this submission and believe that I have an appropriate level of expertise to confirm that it is of an acceptable scientific standard.

# Peer review: Reviewer response for version 1

**DOI:** 10.5256/f1000research.29393.r77029

**Published:** 2021-02-04

**Authors:** Ronell Bologna-Molina

**Affiliations:** 1 Molecular Pathology Area, School of Dentistry, Universidad de la República, Montevideo, Uruguay; 2 Universidad Juárez del Estado de Durango, Durango, Mexico

It is a clear, simple and interesting article, easily understood by the reader. The authors describe a rare case of an odontoma in the anterior left side of the maxilla associated with an impacted canine and supernumerary tooth with a governmental canal.

 There are some points that could improve the quality of the article:

- a) The x-ray image is not clearly representative of a composite odontoma, it could also be a complex odontoma. Due to the aforementioned, I suggest that in the histopathological image 4a and 4b) the authors mark and mark each dental tissue that makes up the odontoma (dentin, enamel, cementum). In image 4c, the authors should put the following description: It is observed odontogenic epithelium arranged in islands and cords surrounded by mature connective tissue, suggesting the presence of epithelial lamina surrounded by collagenous. Please indicate the magnification of the image (for example 50X, 100X or 400X).
- b) The reviewer suggests (in the discussion section) to go deeper into the imaging of the gubernacular tract with CBCT and the presence of odontomas or odontogenic tumors, I suggest reading in this article: Chaudhry and Sobti (2020 ^1^).
- c) I believe that the conclusion paragraph that says "The dentist should also be aware of the probability of a close relationship between the development of odontoma and the presence of the governmental tract, which in future could be used as a radiographic diagnostic criterion of an odontoma" is very ambitious. I suggest that the authors clearly explain why they believe that it could become a radiographic criterion for an odontoma and/or not for another odontogenic tumor that has the presence of mineralized tissue. 

Minor points:

- In the case report section, the authors describe the patient in the following way: "A 47-year old house wife - of African origin -" I think that this data is not important for the clinical case, I suggest describing only; "A 47-year old female ...".

Are enough details provided of any physical examination and diagnostic tests, treatment given and outcomes?

Yes

Is the case presented with sufficient detail to be useful for other practitioners?

Partly

Is sufficient discussion included of the importance of the findings and their relevance to future understanding of disease processes, diagnosis or treatment?

Partly

Is the background of the case’s history and progression described in sufficient detail?

Yes

Reviewer Expertise:

Oral Pathology, Histopathology,Oral Medicine

I confirm that I have read this submission and believe that I have an appropriate level of expertise to confirm that it is of an acceptable scientific standard, however I have significant reservations, as outlined above.

## References

[1] : Imaging characteristics of Gubernacular Tract on CBCT- A pictorial review. *Oral Radiol*.2020; 10.1007/s11282-020-00461-y 10.1007/s11282-020-00461-y 32607940

# Response

**Published:** 2021-02-13

**Authors:** Lubna Elsayed

**Affiliations:** Princess Nourah bint Abdulrahman University, Saudi Arabia

- ***a)** The x-ray image is not clearly representative of a composite odontoma, it could also be a complex odontoma. Due to the aforementioned, I suggest that in the histopathological image 4a and 4b) the authors mark and mark each dental tissue that makes up the odontoma (dentin, enamel, cementum). In image 4c, the authors should put the following description: It is observed odontogenic epithelium arranged in islands and cords surrounded by mature connective tissue, suggesting the presence of epithelial lamina surrounded by collagenous. Please indicate the magnification of the image (for example 50X, 100X or 400X).*
- **Response a:** thanks for your opinion, it was considered and added (changes were added to the newly submitted version of manuscript).

- ***b)** The reviewer suggests (in the discussion section) to go deeper into the imaging of the gubernacular tract with CBCT and the presence of odontomas or odontogenic tumors, I suggest reading in this article: Chaudhry and Sobti (202 1).*
- **Response b: **Thanks for your recommendation and the suggested paper was very valuable.
- ***c)** I believe that the conclusion paragraph that says "The dentist should also be aware of the probability of a close relationship between the development of odontoma and the presence of the governmental tract, which in future could be used as a radiographic diagnostic criterion of an odontoma" is very ambitious. I suggest that the authors clearly explain why they believe that it could become a radiographic criterion for an odontoma and/or not for another odontogenic tumor that has the presence of mineralized tissue. *
- **Response c:**
  Thanks and a paragraph was added in the discussion to explain the role of GT in differentiating between odontogenic and non-odontogenic lesions based on previous studies.

***Minor points:***

- *In the case report section, the authors describe the patient in the following way: "A 47-year old housewife - of African origin -" I think that this data is not important for the clinical case, I suggest describing only; "A 47-year old female ...".*

**Response for minor point:**

 The recommendation was considered and changes were made, although it was a request by the editor.

# Peer review: Reviewer response for version 1

**DOI:** 10.5256/f1000research.29393.r78510

**Published:** 2021-02-02

**Authors:** Prashanth Panta

**Affiliations:** 1 Department of Oral Medicine and Radiology, MNR Dental College and Hospital, Sangareddy, Telangana, India

Odontoma is a common clinical entity and is often spotted on dental radiographs taken to inspect missing permanent teeth. However, this report's merit lies in the CBCT demonstration of an odontoma within the 'gubernacular canal' (GC)-a finding less frequently spotted on radiographs. Although vital in tooth eruption, GC goes unnoticed in dental radiographs due to a lack of focus on this crucial anatomical landmark. As rightly suggested by few previous authors and the authors of this report, this typical association could aid the radiographic diagnosis of odontoma. Because odontomas are closely associated with GC, they cause delayed/failure of eruption of the underlying permanent tooth. Since many clinicians may still be unaware of some of these aspects, more technical reports are necessary for this direction. The current report by Lubna K. Elsayed *et al.* fills this gap. 

Here are a few recommendations which could improve the manuscript:

1. The 'A 47-year old housewife – of African origin presented' can be modified as 'A 47-year old female – of African origin presented' 
2. Supernumerary tooth and odontoma are also associated with syndromes. If the patient is non-syndromic, the phrase, ' an otherwise healthy and non-syndromic patient,' can be added to the case history section. A brief discussion on the syndromes associated with odontomas and supernumerary teeth may be a valuable addition to this report. 
3. In the conclusion section, the sentence "Thus, early detection and management of odontomas can help correct any dental irregularity and avoid further complications" has been included. When odontomas are asymptomatic entities and discovered incidentally during radiographic surveys taken for evaluation of missing permanent teeth, how is early detection achievable? Consider adding some info regarding the same and mention which are the further complications. 
4. Since the report mainly circles around GC, the conclusion paragraph could straightaway focus on the GC-odontoma connection. Consider avoiding conventional odontoma literature in conclusion. The below paragraph seems to be fitting:
*'The dentist should be aware of the probability of a close relationship between the development of odontoma and presence of the gubernacular tract, which could be used as a radiographic diagnostic criterion of an odontoma.' *
5. The discussion section can also include a brief review of *odontoma-GC literature* and *appropriate differential diagnosis.  *
6. To avoid hype, the word 'unusual' can be removed from the title. The title *'Association of the gubernacular canal, supernumerary tooth, and odontoma with an impacted canine on cone-beam computed tomography'* seems fitting.  

Are enough details provided of any physical examination and diagnostic tests, treatment given and outcomes?

Yes

Is the case presented with sufficient detail to be useful for other practitioners?

Yes

Is sufficient discussion included of the importance of the findings and their relevance to future understanding of disease processes, diagnosis or treatment?

Yes

Is the background of the case’s history and progression described in sufficient detail?

Yes

Reviewer Expertise:

Oral medicine and radiology.

I confirm that I have read this submission and believe that I have an appropriate level of expertise to confirm that it is of an acceptable scientific standard, however I have significant reservations, as outlined above.

# Response

**Published:** 2021-02-13

**Authors:** Lubna Elsayed

**Affiliations:** Princess Nourah bint Abdulrahman University, Saudi Arabia

- ***Recommendation 1**: The 'A 47-year old housewife – of African origin presented' can be modified as 'A 47-year old female – of African origin presented' *

**Answer: **Thanks for the recommendation and the change is accepted, although it was a request of the editor to mention “occupation”.

- ***Recommendation 2:** Supernumerary tooth and odontoma are also associated with syndromes. If the patient is non-syndromic, the phrase, 'an otherwise healthy and non-syndromic patient,' can be added to the case history section. A brief discussion on the syndromes associated with odontomas and supernumerary teeth may be a valuable addition to this report.*

**Answer:** Thanks for your opinion, it was considered.

- ***Recommendation 3:**  In the conclusion section, the sentence "Thus, early detection and management of odontomas can help correct any dental irregularity and avoid further complications" have been included. When odontomas are asymptomatic entities and discovered incidentally during radiographic surveys taken for evaluation of missing permanent teeth, how is early detection achievable? Consider adding some info regarding the same and mention which are the further complications.*

**Answer:** ****Thanks for the suggestion and further complications were added to the discussion section

- ***Recommendation 4**: Since the report mainly circles around GC, the conclusion paragraph could straightaway focus on the GC-odontoma connection. Consider avoiding conventional odontoma literature in conclusion. *

**Answer: **Thanks for the valuable comment and the recommended phrase was added to the conclusion section.

- ***Recommendation 5:**The discussion section can also include a brief review of odontoma-GC literature and appropriate differential diagnosis. *

**Answer: **Thanks for the comment and the suggestion was considered and added to the discussion section.

- ***Recommendation 6: **To avoid hype, the word 'unusual' can be removed from the title. The title 'Association of the gubernacular canal, supernumerary tooth, and odontoma with an impacted canine on cone-beam computed tomography' seems fitting.
**  ***

**Answer: **Thanks for the suggestion and it was considered.

# Peer review: Reviewer response for version 1

**DOI:** 10.5256/f1000research.29393.r72618

**Published:** 2020-11-17

**Authors:** Hatem W. Amer

**Affiliations:** 1 Oral and Maxillo-facial Pathology Department, Faculty of Dentistry, Cairo University, Cairo, Egypt

The introduction is well written. The methodology and approach to diagnosis was systematic and the discussion was fruitful. It would have been more fulfilling to provide more past literature about the case and to discuss how the gubernacular canal could affect differential diagnosis both radiographically and histologically. All in all I found it very interesting.

Are enough details provided of any physical examination and diagnostic tests, treatment given and outcomes?

Yes

Is the case presented with sufficient detail to be useful for other practitioners?

Yes

Is sufficient discussion included of the importance of the findings and their relevance to future understanding of disease processes, diagnosis or treatment?

Yes

Is the background of the case’s history and progression described in sufficient detail?

Yes

Reviewer Expertise:

Oral and maxillofacial pathology

I confirm that I have read this submission and believe that I have an appropriate level of expertise to confirm that it is of an acceptable scientific standard.

# Response

**Published:** 2021-02-13

**Authors:** Lubna Elsayed

**Affiliations:** Princess Nourah bint Abdulrahman University, Saudi Arabia

Thank you for your valuable comments and we appreciate your recommendations, and they were considered, a brief description about how the gubernacular canal could affect differential diagnosis both radiographically and histologically was added to the discussion section.

# Response

**Published:** 2021-02-23

**Authors:** Lubna Elsayed

**Affiliations:** Princess Nourah bint Abdulrahman University, Saudi Arabia

Thank you for your valuable comments, and your recommendations were considered as we added more literature about similar cases and a discussion about how the gubernacular canal could affect differential diagnosis both radiographically and histologically was added in the discussion section.
